# Unilateral Biportal Endoscopic Laminectomy for Treating Cervical Stenosis: A Technical Note and Preliminary Results

**DOI:** 10.3390/medicina59020305

**Published:** 2023-02-07

**Authors:** Chengyue Zhu, Xizhuo Zhou, Guofen Ge, Cuijuan Wang, Xiaoshan Zhuang, Wei Cheng, Dong Wang, Hang Zhu, Hao Pan, Wei Zhang

**Affiliations:** 1 Department of Orthopaedics, Hangzhou Traditional Chinese Medicine Hospital Affiliated to Zhejiang Chinese Medical University, Tiyuchang Road NO 453, Hangzhou 310007, China; 2 Hangzhou School of Clinical Medicine, Zhejiang Chinese Medical University, Binwen Road NO 548, Hangzhou 310053, China; 3 Department of Orthopaedics, Hangzhou Dingqiao Hospital, Huanding Road NO 1630, Hangzhou 310021, China

**Keywords:** unilateral biportal endoscopy, cervical stenosis, laminectomy, minimally invasive spine surgery

## Abstract

*Objective*: The objective of this study was to introduce a surgical technique for the percutaneous decompression of cervical stenosis (CS) using a unilateral biportal endoscopic approach and characterize its early clinical and radiographic results. *Materials and Methods*: Nineteen consecutive patients with CS who needed surgical intervention were recruited. All enrolled patients underwent unilateral biportal endoscopic laminectomy (UBEL). All patients were followed postoperatively for >1 year. The preoperative and final follow-up evaluations included the Japanese Orthopedic Association (JOA) score for neurological assessment, visual analogue scale (VAS) for axial pain and C2–C7 Cobb angle for cervical sagittal alignment. The postoperative complications were analyzed. *Results*: Thirteen males and six females were included in the analysis. The mean follow-up period was 16.3 ± 2.6 months. The mean operative time was 82.6 ± 18.4 min. Postoperative MRI and CT revealed ideal neural decompression of the treated segments in all patients. Preoperative VAS and JOA scores improved significantly after the surgery, and cervical lordosis was preserved on the postoperative images. *Conclusions*: UBEL was an effective surgical method for CS, which may also minimize iatrogenic damage to the posterior tension band (PTB) and help to maximize the preservation of the cervical lordosis.

## 1. Introduction

CS is often caused by a pincer mechanism, and common pathogenic factors include protruded intervertebral discs, ossification of the posterior longitudinal ligament and hypertrophic ligamentum flavum. Anterior cervical decompression and fusion (ACDF) is a common method due to its ability to directly remove the compressive lesions and perform a muscle-sparing dissection, which results in minor postoperative pain and faster recovery [1]. However, the loss of motion segment and adjacent segmental degeneration cannot be underestimated [2]. Conventional expansive laminoplasty and laminectomy have shown favorable clinical outcomes despite the disadvantages of persistent axial pain and progressive cervical kyphosis [3,4].

Minimally invasive laminectomy has become increasingly popular for the treatment of CS, which can allow for multilevel decompression with the preservation of the posterior muscle ligament complex [5]. Nevertheless, full endoscopic laminectomy, as well as microendoscopic laminectomy, require a steep learning curve and specially designed instruments limit their wide application [6,7]. Unilateral biportal endoscopy (UBE) is a newly developed minimally invasive spine surgery that can be conducted with the help of a tool kit for arthroscopy and conventional instruments [8]. Compared with full endoscopic/microendoscopic laminectomy, UBE laminectomy (UBEL) can be achieved without the use of the risky “over the top” technique. The aim of this study was to introduce the surgical technique for UBEL and present preliminary clinical and radiologic outcomes.

## 2. Materials and Methods

### 2.1. Ethics Statement and Subjects

The study was approved by our institutional review board (No. 202007102020000214122). Informed consent was obtained from all the patients. Nineteen patients with CS were treated with UBEL between October 2020 and October 2021.

The inclusion criteria for UBEL were as follows: (1) symptomatic CS with at least one clinical sign of myelopathy, (2) CS demonstrated on magnetic resonance imaging (MRI) and computed tomography (CT) scans, (3) >18 years old and (4) refractory to more than three months of conservative treatment.

The exclusion criteria were as follows: (1) segmental instability or cervical kyphosis, (2) previous posterior surgical intervention, (3) decompression > 3 segments and (4) concomitant lumbar stenosis and other pathological conditions, including infection, trauma or tumor.

General status, operative details and radiological images were reviewed (Table 1). Functional outcomes were evaluated using the Japanese Orthopedic Association (JOA) scoring system, and axial neck pain was evaluated using visual analogue scale (VAS). The C2–C7 Cobb angle was calculated by measuring between the C2 and C7 inferior endplates at the neutral position (Table 2).

### 2.2. Surgical Procedure

#### 2.2.1. Position, Incision and Instruments

**Figure 1:**
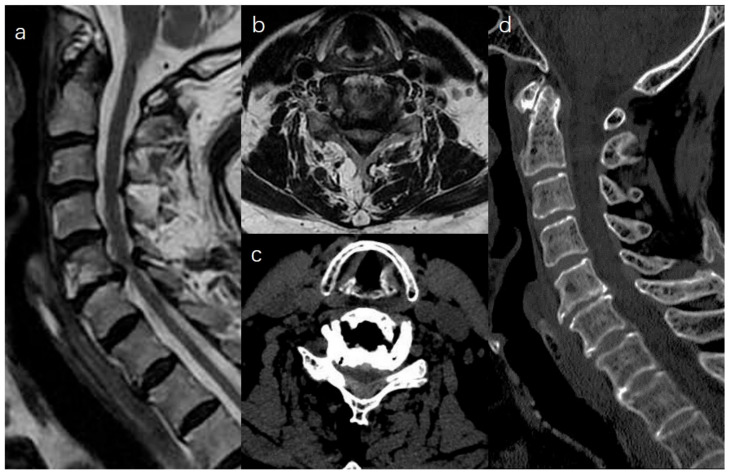
Preoperative MRI showing central canal stenosis at C5–C6 (**a**,**b**). The spinal cord was compressed by a herniated disc and hypertrophied LF on CT scan (**c**,**d**).

C5–C6 decompression is used as an example (Figure 1). Following satisfactory general anesthesia, the patient was placed in the prone position, and the head was secured in a horseshoe headrest. The neck was mildly flexed and fixed using tape.

**Figure 2:**
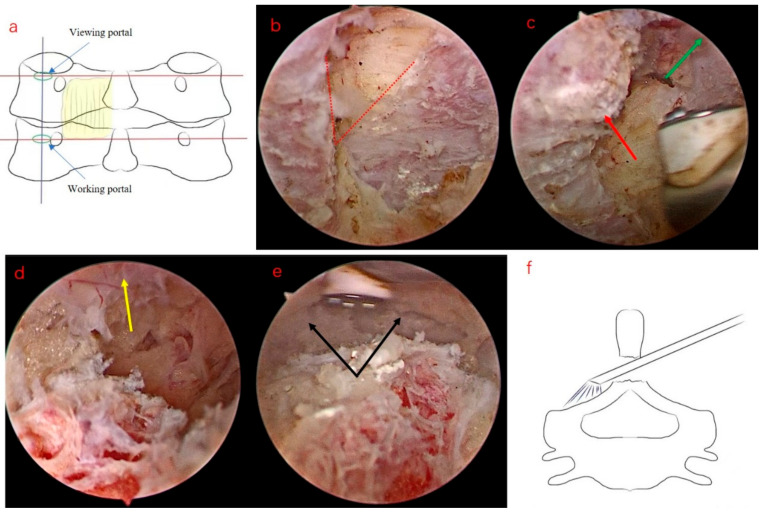
(**a**) Schematic representation of the location of the portals. (**b**–**d**) Intraoperative photographs: “V” point (dotted lines), C5 SP (red arrow), C6 SP (green arrow) and the spared tip of C5 SP (yellow arrow). (**e**) The contralateral laminae were easily exposed (black arrows). (**f**) After the tip of the SP was spared, the endoscope could easily reach the contralateral side.

Two horizontal lines were drawn along the C5 and C6 pedicles, and a vertical line was drawn in the midline of the left lateral mass in the anteroposterior view. The junctional point on the left side served as a viewing portal, while the other on the right side served as a working portal (Figure 2a).

A high-speed diamond burr, specially designed arthroscopic facilities, a tool-kit of radiofrequency (RF) systems (Jiangsu BONSS Medical Technology, Taizhou, China) and conventional spine surgical instruments, including pituitary forceps, mini bush-hooks and Kerrison rongeurs, were used.

#### 2.2.2. Unilateral Biportal Approach for Bilateral Laminectomy (Video S1)

**Figure 3:**
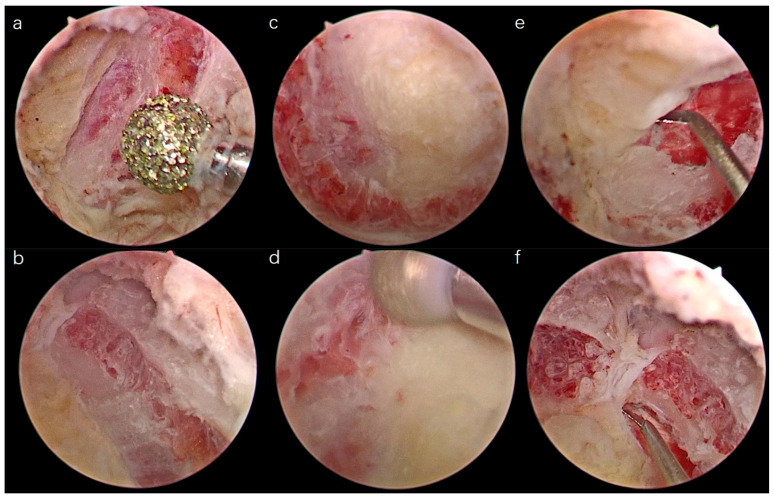
The ipsilateral and contralateral inferior insertion of the LF was exposed (**a**,**b**). The ipsilateral and contralateral superior insertion of the LF was exposed (**c**,**d**). The mini bush-hook was used to liberate the LF bilaterally (**e**,**f**).

**Figure 4:**
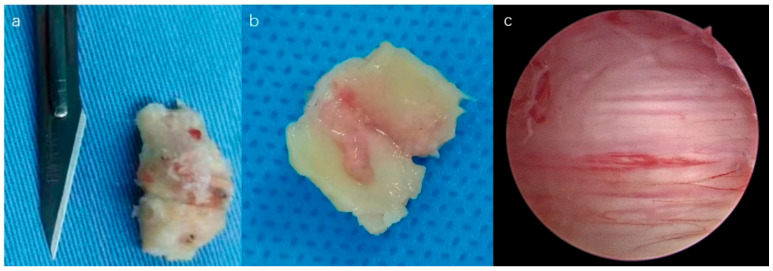
The dorsal and ventral surface of LF (**a**,**b**). The cord was fully decompressed (**c**).

First, an RF was used to expose the intersection of the C5–C6 laminae and facet joint, namely, the “V” point (Figure 2b). Spinous process (SP) of C5–C6 was also exposed (Figure 2c), and the tip of the SP of C5 was cut using a 4 mm diamond burr (Figure 2d,f). The tip was floated automatically, and a wide field was visualized (Figure 2e). The paraspinal muscle was detached from the contralateral C5–C6 laminae. Second, a 4 mm high-speed burr was used to drill the lower part of the C5 lamina and upper part of the C6 lamina until the cephalad and caudad entheses of the ligamentum flavum (LF) was exposed (Figure 3a–d). A longitudinal cut on the lateral part of the LF was performed bilaterally with a mini bush-hook (Figure 3e,f), and the floated ligamentum flavum was observed (Figure 4a,b) and removed carefully. Finally, a scrupulous hemostasis was conducted, a drainage tube was inserted when dural pulsation was confirmed (Figure 4c), and incisions were sutured using a standard method.

If multilevel laminectomy was needed, the endoscope and instruments were slid either cranially or caudally, and decompression was performed identically.

## 3. Statistical Analysis

Student’s *t*-test was used to compare the JOA scores, VAS and C2–C7 Cobb angle pre- and postoperatively. All statistical analyses were performed using SPSS version 21, and the statistical significance was set at *p*  <  0.05.

## 4. Results

The patient cohort consisted of 13 men and 6 women with an average age of 65.2 ± 7.7 years. One patient who had radiculopathy in addition to myelopathy received a foraminotomy operation. The mean operation time was 82.6 ± 18.4 min, the mean estimated blood loss was 38.4 ± 19.2 mL, and the mean hospital stay was 2.6 ± 1.2 days. The average follow-up time for the patients was 16.3 ± 2.6 months. The mean JOA score was 10.8 ± 1.5 preoperatively and improved to 13.9 ± 1.0 at the final follow up (*p* = 0.000), and the mean VAS score was 2.3 ± 0.7 preoperatively and improved to 2.1 ± 0.5 postoperatively (*p* = 0.042). Radiographically, the mean C2–C7 Cobb angle was 14.8 ± 5.5° preoperatively and 15.3 ± 5.4° at the last follow-up visit (*p* = 0.439). A resultant paralysis of the epidural hematoma was encountered 2 h after the operation, and the patient was treated with UBE revision and achieved a full recovery. No permanent neurological deficits occurred in this cohort.

## 5. Discussion

To the best of our knowledge, this is the first report to describe the UBEL technique.

**Figure 5:**
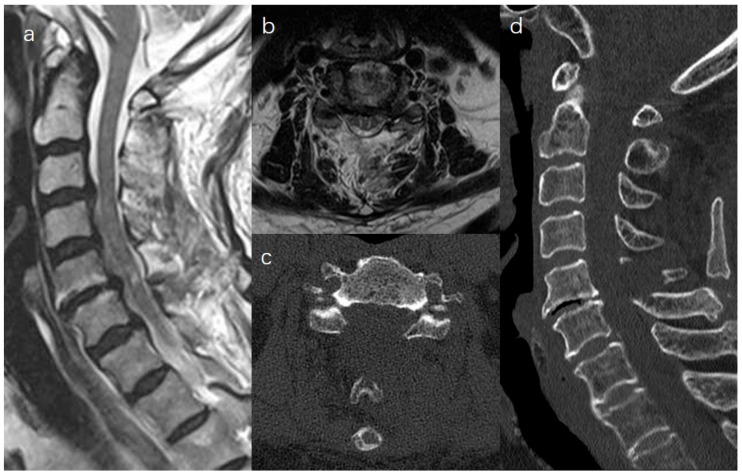
Postoperative MRI showed hypertrophied LF was removed (**a**,**b**). A CT scan revealed that laminectomy was performed, while PTB and cervical lordosis were preserved (**c**,**d**).

Endoscopic laminectomy with minimal muscle invasion was achieved. The patients’ clinical symptoms were significantly improved, and loss of lordosis was not observed (Figure 5).

Cervical myelopathy caused by spinal stenosis often requires surgery with a posterior approach. Complications such as axial pain and kyphotic deformity after conventional laminectomy and laminoplasty have been reported due to extensive paraspinal muscle release and retraction [9,10]. In percutaneous full endoscopic and microscopic surgery, the base of the SP must be generously removed if unilateral laminotomy with bilateral decompression is needed, which may result in much more intraspinal work and higher risk [6]. Meanwhile, operation and observation in the same single portal proved less facilitation [11].

The UBE technique has been widely applied in the treatment of degenerative diseases of the lumbar spine, with the advantages of flexible manipulation and a wide operation field [12]. The application of UBE in the cervical spine was mainly in foraminal stenosis [13]. Kim reported a UBE technique to decompress central canal stenosis, but the technical requirements were relatively demanding [14]. The authors not only designed a contralateral “Zhang’s portal” to facilitate single level decompression [15] but also modified an endoscopic expansive laminoplasty for multisegmental stenosis [16]. In addition, we attempted UBEL combined with the lateral mass screw fixation technique to treat CS with instability [17]. However, additional iatrogenic injury needs to be considered in the abovementioned techniques.

UBEL led to favorable clinical results due to its minimal invasiveness. All patients showed improved JOA and VAS scores and were satisfied with slight postoperative neck pain, small operative scars and short hospital stays. The following characteristics of UBEL might have given rise to such successful results in these series. First, a wide surgical field was provided when the tip of the SP was cut off and then floated with the help of the traction of the supraspinous ligament and buoyancy of the irrigation fluid. The muscle was easily detached from the bilateral laminae using a radiofrequency probe, and the bilateral junctions of the laminar and lateral mass could be identified distinctly. Thus, invasion of the facet complex was avoided, which prevented postlaminectomy kyphosis. Second, the cervical laminae are arranged very closely, and the RF can be slid laminar by laminar to safely expose the surgical field. Consequently, multilevel decompression can be achieved through two portals without auxiliary portals. Additionally, conventional surgical instruments of a large size make it more efficient for the surgeon to perform complicated operations.

There have been no long-term follow-up studies on the clinical and radiologic results of UBEL in the treatment of CS, particularly the development of sagittal imbalance. In a two-year follow-up study, conventional laminectomy with instrumented fusion and laminectomy alone in degenerative cervical myelopathy (DCM) revealed similar effectiveness [18]. Dios compared patient-reported 5-year clinical results between conventional laminectomy alone and laminectomy with instrumented fusion in patients with DCM and found no important differences [19]. A recent narrative review concluded that conventional laminectomy alone was an effective treatment without the additional risks of posterior instrumentation in highly selected patients with normal preoperative cervical sagittal alignment [20]. Hence, UBEL, which preserves dorsal structures, including muscles and ligaments, has theoretical advantages in reducing the risk of progression to postoperative cervical kyphosis. Given the growing evidence of the importance of cervical lordosis and its impact on functional and neurological outcomes, the ability of the UBEL to maintain lordosis will become even more important as we follow these patients for a longer period.

We reported a 53-year-old male with paralysis due to postoperative cervical epidural hematoma after a three-level flavectomy. Previous research demonstrated an incidence of 24.7% radiological postoperative spinal epidural hematoma (PSEH) in the UBE decompression of lumbar stenosis; however, only 1.2% of patients received revision surgery due to neurological deficits [21]. Amiri reported 0.22% symptomatic PSEH among 4568 patients who underwent open spinal surgeries and elucidated that alcohol consumption > 10 U/w, previous spinal procedures and multilevel procedures were significant risk factors for PSEH [22]. Multilevel UBEL in the cervical spine involves plenty of bony work, and operators always concentrated on stopping the bleeding from intracanal vein plexus and oozing from the bone surface, but adequate muscle hemostasis in the portals deserves more attention. We also assume that pressure inside the artificial surgical space masks the bleeding spots and results in hematoma. The patient underwent a UBE revision surgery for evacuation of the epidural hematoma and showed immediate improvement. He did not have neurological symptoms at the end of the last follow-up.

There were some limitations of this procedure, including the small sample size and short follow-up period. It is noteworthy that although UBEL carries the advantages of other endoscopic techniques, it is not applicable to CS patients, such as those who have cervical kyphosis, those who have more than three-level stenosis or segmental listheses and those for whom dynamic imaging indicates instability. This study must strictly be interpreted as describing a novel approach for a selected group of CS patients. More evidence of clinical outcomes will be provided by additional studies comparing this method to other posterior approach procedures for CSM, such as conventional open laminectomies and laminoplasties and full endoscopic and microendoscopic laminectomies.

## 6. Conclusions

This work demonstrates that UBEL may be considered an excellent surgical alternative to treat CS without the development of iatrogenic kyphosis. It represents an effective method with neurological and radiological outcomes with less soft tissue invasion, which translates into dramatically less postoperative axial pain and maintained postoperative cervical lordosis.

## Figures and Tables

**Table 1:** Patient characteristics.

Characteristic	Value
Age, years	65.2 ± 7.7
Sex, male/female	13/6
Operation time, minutes	82.6 ± 18.4
Estimated blood loss, ml	38.4 ± 19.2
Level of flavectomy	
C4/5	3
C5/6	6
C6/7	6
C5-7	2
C3-6	2
Hospital stay	2.6 ± 1.2
Surgical complication	
Epidural hematoma	1
Follow-up periods, month	16.3 ± 2.6

**Table 2:** Pre- and postoperative outcomes.

Outcomes	Preoperative	Final Follow-Up	*p* Value
JOA	10.8 ± 1.5	13.9 ± 1.0	0.000
VAS of axial pain	2.3 ± 0.7	2.1 ± 0.5	0.042
C2–C7 Cobb angle	14.8 ± 5.5	15.3 ± 5.4	0.439

## Data Availability

The original contributions presented in the study are included in the article/Supplementary Material, further inquiries can be directed to the corresponding author.

## Supplementary Materials

The following supporting information can be downloaded at: https://www.mdpi.com/article/10.3390/medicina59020305/s1, Video S1: Unilateral biportal endoscopic laminectomy for cervical stenosis.
